# Supplementary material for: Development of chemokine network inhibitors using combinatorial saturation mutagenesis
Source: Commun Biol. 2025 Apr 3;8:549. doi: 10.1038/s42003-025-07778-6 (PMC11969024; doi:10.1038/s42003-025-07778-6)
Supplement: Supplementary file 4 — Supplementary Data 2 [file 42003_2025_7778_MOESM4_ESM.zip › SupplementaryData3/scripts/xcomb.tar!/./script/analysis/JK/HD2XC_allheatmap.pdf]

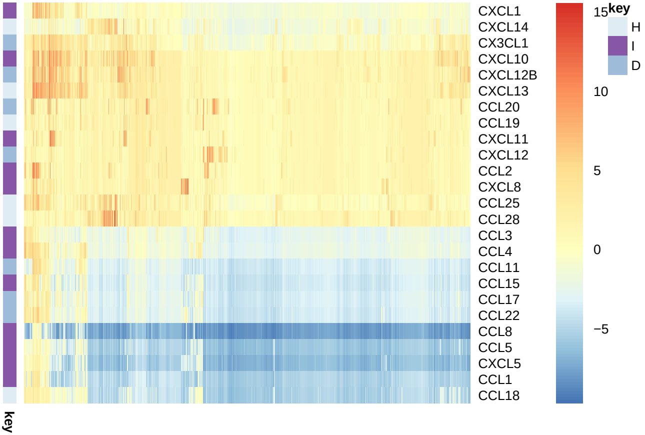

CXCL1
CXCL14
CX3CL1
CXCL10
CXCL12B
CXCL13
CCL20
CCL19
CXCL11
CXCL12
CCL2
CXCL8
CCL25
CCL28
CCL3
CCL4
CCL11
CCL15
CCL17
CCL22
CCL8
CCL5
CXCL5
CCL1
CCL18
15
10
5
0
−5
key
H
I
D
key
